# Supplementary material for: Prediction of future visceral adiposity and application to cancer research: The Multiethnic Cohort Study
Source: PLoS One. 2024 Jul 18;19(7):e0306606. doi: 10.1371/journal.pone.0306606 (PMC11257330; doi:10.1371/journal.pone.0306606)
Supplement: S1 Fig — (DOCX) [file pone.0306606.s001.docx]

**S1 Fig. Diagram showing the data sources for current analysis within the Multiethnic Cohort Study.**

The Multiethnic Cohort Study (MEC)

present

1993-1996

MEC is an ongoing prospective follow-up study of surviving baseline participants (n=215,915 in 1993-1996) with annual linkage to SEER registries to identify incident cancer cases and linkage to the National Death Index to verify vital status.

**applying VAT**

**prediction scores**

**VAT**

**prediction modeling**

incident cancer (2001-2013) case-control analysis

n=950 pairs breast cancer

n=831 pairs colorectal cancer

n=831 colorectal cancer

subset (n=500)

APS

(n=1861, 2013-2016)

subset (n=500)

pre-diagnostic

blood

Biorepository

(n~70,000,

2001-2006)
