# Supplementary material for: Prediction of future visceral adiposity and application to cancer research: The Multiethnic Cohort Study
Source: PLoS One. 2024 Jul 18;19(7):e0306606. doi: 10.1371/journal.pone.0306606 (PMC11257330; doi:10.1371/journal.pone.0306606)
Supplement: S1 Table — (DOCX) [file pone.0306606.s002.docx]

**S1 Table. List of measured and derived blood biomarkers used for refitting prediction models** **(previously published in Le Marchand et al. 2020).**

| **Biomarkers** | **Specimen** | **Assay** | **Assay Specifics** | **QC** | |
| --- | --- | --- | --- | --- | --- |
|  |  |  |  |  |  |
|  |  |  |  | **%CV^a^** | **%ICC^a^** |
| **Alanine transaminase** (ALT), U/L | serum | Cobas | Randox Laboratories, AL1205 | 4.4% | 82% |
| **C-reactive protein** (CRP), mg/L | serum | Cobas | Core Lab Supplies, C7568 | 13.0% | 88% |
| **Cholesterol total**, mg/dL | serum | Cobas | Pointe Scientific, H7510 | 1.1% | 64% |
| **Cholesterol high-density lipoprotein** (HDLC), mg/dL | serum | Cobas | Pointe Scientific, H7545 | 1.7% | 78% |
| **Cholesterol low-density lipoprotein** (LDLC), mg/dL | serum | Derived | from cholesterol, HDLC and TG | 2.2% | 62% |
| **Glucose**, mg/dL | serum | Cobas | Randox Laboratories, GL1611 | 0.9% | 86% |
| **Triglycerides** (TG), mg/dL | serum | Cobas | Pointe Scientific, T7532 | 1.8% | 92% |
| **Adiponectin total**, ng/mL | serum | ELISA | R&D, DRP300 | 0.7% | 98% |
| **HOMA-IR** |  | derived |  |  |  |
| **HOMA-beta** |  | derived |  |  |  |
| **Insulin**, microU/mL | serum | ELISA | EMD Millipore, EZH1-14K | 1.8% | 95% |
| **Insulin-like growth factor-1** (IGF1), ng/mL | serum | ELISA | R&D, DG100 | 1.4% | 89% |
| **Insulin-like growth factor binding protein-1** (IGFBP1), ng/mL | serum | ELISA | R&D, DGB200 | 6.4% | 86% |
| **Insulin-like growth factor binding protein-2** (IGFBP2), ng/mL | serum | ELISA | R&D, DGB200 | 1.2% | 97% |
| **Insulin-like growth factor binding protein-3** (IGFBP3), ng/mL | serum | ELISA | R&D, DGB300 | 0.7% | 99% |
| **Leptin** , ng/mL | serum | ELISA | R&D, DLP00 | 2.5% | 94% |
| **Lipopolysaccharide binding protein** (LBP), ng/mL | plasma | ELISA | Cell Sciences, CKH113 | 0.7% | 80% |
| **Sex hormone binding globulin** (SHBG), nmol/L | serum | ELISA | R&D, DSHBG0B | 1.9% | 93% |
| **Lipid-soluble micronutrients**, ng/mL | serum | HPLC [1] |  |  |  |
| **Carotene cis-beta** | serum | HPLC |  | 5.2% | 57% |
| **Carotene trans-alpha** | serum | HPLC |  | 2.0% | 97% |
| **Carotene trans-beta** | serum | HPLC |  | 1.3% | 98% |
| **Cryptoxanthin cis-beta** | serum | HPLC |  | 2.0% | 92% |
| **Cryptoxanthin trans-alpha** | serum | HPLC |  | 2.0% | 90% |
| **Cryptoxanthin trans-beta** | serum | HPLC |  | 1.1% | 99% |
| **Lutein cis1** | serum | HPLC |  | 1.6% | 94% |
| **Lutein cis2** | serum | HPLC |  | 4.1% | 75% |
| **Lutein cis-anhydro** | serum | HPLC |  | 1.5% | 94% |
| **Lutein trans** | serum | HPLC |  | 1.4% | 95% |
| **Lutein trans-anhydro** | serum | HPLC |  | 1.3% | 96% |
| **Lycopene dihydro** | serum | HPLC |  | 2.2% | 79% |
| **Lycopene total** | serum | HPLC |  | 1.1% | 89% |
| **Retinol** | serum | HPLC |  | 1.2% | 89% |
| **Tocopherol alpha** | serum | HPLC |  | 0.6% | 89% |
| **Tocopherol beta+gamma** | serum | HPLC |  | 0.7% | 95% |
| **Tocopherol delta** | serum | HPLC |  | 1.0% | 83% |
| **Tocopherol total** | serum | HPLC |  | 1.1% | 12% |
| **Ubiquinone (oxidized Coenzyme Q10)** | serum | HPLC |  | 3.1% | 76% |
| **Ubiquinol (reduced Coenzyme Q10)** | serum | HPLC |  | 8.8% | 81% |
| **Vitamin D 25-hydroxyvitamin D3** | serum | HPLC |  | 2.9% | 83% |
| **Zeaxanthin** | serum | HPLC |  | 2.1% | 93% |
| **Sex steroid hormones, pg/mL** | serum | LC/MS [2, 3] |  |  |  |
| **Estradiol unconjugated** | serum | LC/MS |  | 19.9% | 94% |
| **Estradiol free** | serum | Derived | from unconjugated estradiol & SHBG [4] | 24.4% | 90% |
| **Estrone unconjugated** | serum | LC/MS |  | 9.3% | 87% |
| **Estrone free** | serum | Derived | from unconjugated estrone & SHBG | 15.7% | 85% |
| **Testosterone unconjugated** | serum | LC/MS |  | 2.1% | 97% |
| **Testosterone free** | serum | Derived | from unconjugated testosterone & SHBG | 6.1% | 99% |

Abbreviations: CV (coefficient of variation), ELISA (enzyme-linked immunosorbent assay), HPLC (high-pressure liquid chromatography), ICC (intra-class correlation coefficient), LC/MS (liquid chromatography/mass spectrometry).

^a^ Approximately 10% blinded QC samples were inserted among study samples at random locations and analyzed to obtain CV (from all QC replicates) and ICC (from QC replicates included in multiple batches).
