# Supplementary material for: Prediction of future visceral adiposity and application to cancer research: The Multiethnic Cohort Study
Source: PLoS One. 2024 Jul 18;19(7):e0306606. doi: 10.1371/journal.pone.0306606 (PMC11257330; doi:10.1371/journal.pone.0306606)
Supplement: S2 Table — (DOCX) [file pone.0306606.s003.docx]

**S2 Table. The original VAT prediction score equation to predict concurrent VAT in the Adiposity Phenotype Study (published in Le Marchand et al. 2020).**

|  | **Beta in log units** | |
| --- | --- | --- |
|  | **Men** | **Women** |
| **Intercept** | -21.4689 | -13.2449 |
| **BMI (kg/m^2^)** | 13.7906 | 9.4543 |
| **Adiponectin (ng/mL)** | -0.0362 | -0.0686 |
| **HDL cholesterol (mg/dL)** | 0.0815 | 0.0489 |
| **LDL cholesterol (mg/dL)** | 0.2065 | 0.2477 |
| **Total cholesterol (mg/dL)** | -0.2859 | -0.4467 |
| **Insulin (microU/mL)** | 0.0859 | 0.1141 |
| **Leptin (ng/mL)** | 0.0690 | 0.0089 |
| **Triglycerides (mg/dL)** | 0.1344 | 0.2492 |
| **Total carotene (ng/mL)** | -0.0724 | -0.0448 |
| **Sex hormone-binding globulin (nmol/L)** | -0.0975 | -0.0483 |
| **Height (m)** | 5.4779 | 2.9285 |
| **BMI squared (kg^2^/m^4^)** | -1.8282 | -1.2019 |
| **Height squared (m^2^)** | -5.0420 | -3.3853 |

The original concurrent VAT prediction equation was, for men:

ln(VAT) = -21.4689 + 13.7906*ln(BMI) – 0.0362*ln(adiponectin) +0.0815*ln(HDL cholesterol) + 0.2065*ln(LDL cholesterol) – 0.2859*ln(total cholesterol) + 0.0859*ln(insulin) + 0.0690*ln(leptin) + 0.1344*ln(triglycerides) - 0.0724*ln(total carotene) - 0.0975*ln(sex hormone-binding globulin) + 5.4779*ln(height) - 1.8282*(ln(BMI))^2^ - 5.0420*(ln(height))^2^

and for women:

ln(VAT) = -13.2449 + 9.4543*ln(BMI) – 0.0686*ln(adiponectin) +0.0489*ln(HDL cholesterol) + 0.2477*ln(LDL cholesterol) – 0.4467*ln(total cholesterol) + 0.1141*ln(insulin) + 0.0089*ln(leptin) + 0.2492*ln(triglycerides) - 0.0448*ln(total carotene) - 0.0483*ln(sex hormone-binding globulin) + 2.9285*ln(height) - 1.2019*(ln(BMI))^2^ - 3.3853*(ln(height))^2^
