# Supplementary material for: Prediction of future visceral adiposity and application to cancer research: The Multiethnic Cohort Study
Source: PLoS One. 2024 Jul 18;19(7):e0306606. doi: 10.1371/journal.pone.0306606 (PMC11257330; doi:10.1371/journal.pone.0306606)
Supplement: S3 Table — (DOCX) [file pone.0306606.s004.docx]

**S3 Table. Averaged elastic net regression coefficients for the refit prediction models.**

|  | **Men (n=235)** | | **Women (n=244)** | |
| --- | --- | --- | --- | --- |
| **Variables** | **Beta in log units** | **Standardized Beta** | **Beta in log units** | **Standardized Beta** |
| **Intercept** | 0.858701027 | 5.218936163 | -14.51641569 | 4.742846649 |
| **Height at QX1 (m)** | -0.859154857 | -0.037584911 | 9.244076474 | 0.41581691 |
| **Height squared (m^2^)** | 0 | 0 | -9.659591795 | -0.418211082 |
| **BMI at QX3 (kg/m^2^)** | 1.43616609 | 0.22213852 | 9.613875342 | 1.64813122 |
| **BMI squared (kg^2^/m^4^)** | 0 | 0 | -1.273442337 | -1.436022697 |
| **Adiponectin (ng/mL)** | -0.039553517 | -0.024463648 | -0.089481571 | -0.057578602 |
| **ALT (U/L)** | 0.091041495 | 0.038679033 | 0.085430897 | 0.042835983 |
| **HDL cholesterol (mg/dL)** | 0 | 0 | -0.187638897 | -0.073039172 |
| **LDL cholesterol (mg/dL)** | -0.002326523 | -0.000762167 | 0.020136243 | 0.00549378 |
| **Total cholesterol (mg/dL)** | 0 | 0 | 0.034316811 | 0.006755657 |
| **Glucose (mg/dL)** | -0.282829861 | -0.055472311 | 0.053760884 | 0.012196316 |
| **Leptin (ng/mL)** | 0.153448724 | 0.137863285 | 0.051452866 | 0.038319933 |
| **CoQ10 reduced (ng/mL)** | 0.034645109 | 0.037918151 | 0.001141076 | 0.001370905 |
| **Alpha-tocopherol (ng/mL)** | 0.114276165 | 0.043241836 | 0.029647249 | 0.011320715 |
| **Total lutein anhydro (ng/mL)** | 0.023358194 | 0.009016808 | -0.143204389 | -0.054917933 |
| **Total carotene (ng/mL)** | -0.065737454 | -0.050940552 | -0.096283516 | -0.064661244 |
| **Total cryptoxanthin (ng/mL)** | -0.153746932 | -0.095623184 | 0.078925512 | 0.047546832 |
| **VAT score (mean ± SD)** | 5.22 ± 0.40 | | 4.74 ± 0.37 | |
| **R^2^ all** | 0.61 | | 0.51 | |
| **R^2^ by race/ethnicity** |  | |  | |
| **African Americans** | 0.52 | | 0.45 | |
| **Native Hawaiians** | 0.70 | | 0.60 | |
| **Japanese Americans** | 0.71 | | 0.64 | |
| **Latinos** | 0.57 | | 0.59 | |
| **Whites** | 0.65 | | 0.50 | |
| **AUROC for visceral obesity (VAT >150 cm^2^)** | 0.89 | | 0.82 | |
