# Supplementary material for: Prediction of future visceral adiposity and application to cancer research: The Multiethnic Cohort Study
Source: PLoS One. 2024 Jul 18;19(7):e0306606. doi: 10.1371/journal.pone.0306606 (PMC11257330; doi:10.1371/journal.pone.0306606)
Supplement: S4 Table — (DOCX) [file pone.0306606.s005.docx]

**S4 Table. Averaged elastic net regression coefficients for the enhanced prediction models.**

|  | **Men (n=231)** | | **Women (n=239)** | |
| --- | --- | --- | --- | --- |
| **Variables** | **Beta in log units** | **Standardized Beta** | **Beta in log units** | **Standardized Beta** |
| **Intercept** | 0.821946557 | 5.212638552 | -21.55289216 | 4.7406544 |
| **Height (m)** | -0.828610575 | -0.036487486 | 10.60222645 | 0.49000676 |
| **Height squared (m2)** | -0.004767717 | -0.00023537 | -10.51759481 | -0.46774053 |
| **BMI (kg/m2)** | 1.466523446 | 0.22370477 | 13.52356163 | 2.3885106 |
| **BMI squared (kg2/m4)** | 0 | 0 | -1.867107454 | -2.17673251 |
| **Adiponectin** | -0.048969915 | -0.030475933 | -0.08853055 | -0.05713616 |
| **ALT (U/L)** | 0.087926065 | 0.036633557 | 0.086088895 | 0.04391161 |
| **HDL cholesterol (mg/dL)** | 0 | 0 | -0.163426502 | -0.06395722 |
| **LDL cholesterol (mg/dL)** | 0 | 0 | 0.040924567 | 0.01157459 |
| **Total cholesterol (mg/dL)** | 0 | 0 | 0 | 0 |
| **Glucose (mg/dL)** | -0.318801087 | -0.063025775 | 0.041066212 | 0.00945785 |
| **Leptin (ng/mL)** | 0.14107236 | 0.126659573 | 0.055531257 | 0.04170423 |
| **COQ10 reduced (ng/mL)** | 0.029147557 | 0.031920438 | 0.014704604 | 0.01810296 |
| **Alpha-tocopherol (ng/mL)** | 0.151447958 | 0.057485996 | 0.070425773 | 0.02757702 |
| **Total lutein anhydro (ng/mL)** | 0 | 0 | -0.195790998 | -0.07713343 |
| **Total carotene (ng/mL)** | -0.061416039 | -0.046676959 | -0.101505854 | -0.06882362 |
| **Total cryptoxanthin (ng/mL)** | -0.149438462 | -0.092506485 | 0.134815792 | 0.08377805 |
| **HEI-2015 for dairy** | 0.018038263 | 0.043947993 | -0.004825446 | -0.01306211 |
| **HEI-2015 for sodium** | -0.017702333 | -0.053842814 | -0.011050738 | -0.0351104 |
| **HEI-2015 for refined grains** | 0.003539006 | 0.009975951 | -0.026091733 | -0.07253783 |
| **VAT score (mean ± SD)** | 5.21 ± 0.40 | | 4.74 ± 0.39 | |
| **R^2^ all** | 0.62 | | 0.55 | |
| **R^2^ by race/ethnicity** |  | |  | |
| **African Americans** | 0.53 | | 0.50 | |
| **Native Hawaiians** | 0.64 | | 0.58 | |
| **Japanese Americans** | 0.71 | | 0.66 | |
| **Latinos** | 0.61 | | 0.61 | |
| **Whites** | 0.69 | | 0.49 | |
| **AUROC for visceral obesity (VAT >150 cm^2^)** | 0.90 | | 0.85 | |
