# Supplementary material for: Prediction of future visceral adiposity and application to cancer research: The Multiethnic Cohort Study
Source: PLoS One. 2024 Jul 18;19(7):e0306606. doi: 10.1371/journal.pone.0306606 (PMC11257330; doi:10.1371/journal.pone.0306606)
Supplement: S5 Table — Prediction performance is presented for models including BMI alone, BMI and height, the VAT score developed in MEC-APS, the VAI score, and the METS-VF score. (DOCX) [file pone.0306606.s006.docx]

**S5 Table. Prediction of MRI measurement of VAT using concurrent data in all APS participants (n=1,780 with no missing data on predictors).** Prediction performance is presented for models including BMI alone, BMI and height, the VAT score developed in MEC-APS, the VAI score, and the METS-VF score.

|  | **Men (n=874)** | **Women (n=906)** |
| --- | --- | --- |
| **Data/predictor source** | **APS (2013-2016)** | **APS (2013-2016)** |
| **BMI alone** |  |  |
| **R^2^ (AUROC for VAT area >150 cm^2^) in all racial/ethnic groups** | 0.50 (0.87) | 0.48 (0.83) |
| **R^2^ (AUROC) by race/ethnicity** |  |  |
| **African Americans** | 0.32 (0.79) | 0.39 (0.81) |
| **Native Hawaiians** | 0.51 (0.89) | 0.59 (0.84) |
| **Japanese Americans** | 0.64 (0.90) | 0.56 (0.87) |
| **Latinos** | 0.42 (0.87) | 0.53 (0.83) |
| **Whites** | 0.59 (0.88) | 0.57 (0.88) |
| **BMI, BMI^2^, height, and height^2^** |  |  |
| **R^2^ (AUROC) in all racial/ethnic groups** | 0.57 (0.89) | 0.55 (0.82) |
| **R^2^ (AUROC) by race/ethnicity** |  |  |
| **African Americans** | 0.42 (0.80) | 0.41 (0.82) |
| **Native Hawaiians** | 0.58 (0.89) | 0.68 (0.86) |
| **Japanese Americans** | 0.67 (0.90) | 0.63 (0.88) |
| **Latinos** | 0.47 (0.88) | 0.58 (0.83) |
| **Whites** | 0.69 (0.90) | 0.65 (0.90) |
| **VAT score (“Original”) in MEC-APS (Le Marchand 2020)[5]** |  |  |
| **mean (SD)** | 5.20 (0.40) | 4.79 (0.40) |
| **R^2^ (AUROC) in all racial/ethnic groups** | 0.63 (0.90) | 0.68 (0.86) |
| **R^2^ (AUROC) by race/ethnicity** |  |  |
| **African Americans** | 0.47 (0.86) | 0.56 (0.84) |
| **Native Hawaiians** | 0.67 (0.91) | 0.73 (0.85) |
| **Japanese Americans** | 0.72 (0.93) | 0.74 (0.91) |
| **Latinos** | 0.48 (0.86) | 0.67 (0.86) |
| **Whites** | 0.73 (0.93) | 0.69 (0.88) |
| **VAI score (Amato 2010)[6]** |  |  |
| **mean (SD)** | 1.73 (2.21) | 2.02 (2.43) |
| **R^2^ (AUROC) in all racial/ethnic groups** | 0.19 (0.78*) | 0.24 (0.70*) |
| **R^2^ (AUROC) by race/ethnicity** |  |  |
| **African Americans** | 0.04 (0.66) | 0.25 (0.70) |
| **Native Hawaiians** | 0.31 (0.86) | 0.28 (0.69) |
| **Japanese Americans** | 0.17 (0.76) | 0.27 (0.72) |
| **Latinos** | 0.08 (0.68) | 0.16 (0.68) |
| **Whites** | 0.26 (0.84) | 0.17 (0.65) |
| **METS-VF score (Bello-Chavolla 2020)[7]** |  |  |
| **mean (SD)** | 7.21 (0.49) | 6.92 (0.54) |
| **R^2^ (AUROC) in all racial/ethnic groups** | 0.61 (0.90) | 0.58 (0.85) |
| **R^2^ (AUROC) by race/ethnicity** |  |  |
| **African Americans** | 0.33 (0.81) | 0.48 (0.83) |
| **Native Hawaiians** | 0.69 (0.93) | 0.66 (0.85) |
| **Japanese Americans** | 0.73 (0.91) | 0.58 (0.86) |
| **Latinos** | 0.52 (0.88) | 0.63 (0.86) |
| **Whites** | 0.69 (0.91) | 0.63 (0.86) |
