# Supplementary material for: Prediction of future visceral adiposity and application to cancer research: The Multiethnic Cohort Study
Source: PLoS One. 2024 Jul 18;19(7):e0306606. doi: 10.1371/journal.pone.0306606 (PMC11257330; doi:10.1371/journal.pone.0306606)
Supplement: S1 File — (DOCX) [file pone.0306606.s007.docx]

**References for Supporting Information.**

1. Cooney RV, Franke AA, Hankin JH, Custer LJ, Wilkens LR, Harwood PJ, Le Marchand L. Seasonal variations in plasma micronutrients and antioxidants. Cancer Epidemiol Biomarkers Prev. 1995;4(3):207-15. Epub 1995/04/01. PubMed PMID: 7606195.

2. Franke AA, Custer LJ, Morimoto Y, Nordt FJ, Maskarinec G. Analysis of urinary estrogens, their oxidized metabolites, and other endogenous steroids by benchtop orbitrap LCMS versus traditional quadrupole GCMS. Anal Bioanal Chem. 2011;401(4):1319-30. Epub 2011/06/30. doi: 10.1007/s00216-011-5164-3. PubMed PMID: 21713421.

3. Blonder J, Johann DJ, Veenstra TD, Xiao Z, Emmert-Buck MR, Ziegler RG, et al. Quantitation of steroid hormones in thin fresh frozen tissue sections. Anal Chem. 2008;80(22):8845-52. Epub 2008/10/22. doi: 10.1021/ac801402a. PubMed PMID: 18937426.

4. Rinaldi S, Geay A, Dechaud H, Biessy C, Zeleniuch-Jacquotte A, Akhmedkhanov A, et al. Validity of free testosterone and free estradiol determinations in serum samples from postmenopausal women by theoretical calculations. Cancer Epidemiol Biomarkers Prev. 2002;11(10 Pt 1):1065-71. Epub 2002/10/12. PubMed PMID: 12376508.

5. Le Marchand L, Wilkens LR, Castelfranco AM, Monroe KR, Kristal BS, Cheng I, et al. Circulating Biomarker Score for Visceral Fat and Risks of Incident Colorectal and Postmenopausal Breast Cancer: The Multiethnic Cohort Adiposity Phenotype Study. Cancer Epidemiol Biomarkers Prev. 2020;29(5):966-73. Epub 20200304. doi: 10.1158/1055-9965.EPI-19-1469. PubMed PMID: 32132150; PubMed Central PMCID: PMCPMC7196505.

6. Amato MC, Giordano C, Galia M, Criscimanna A, Vitabile S, Midiri M, et al. Visceral Adiposity Index: a reliable indicator of visceral fat function associated with cardiometabolic risk. Diabetes Care. 2010;33(4):920-2. Epub 20100112. doi: 10.2337/dc09-1825. PubMed PMID: 20067971; PubMed Central PMCID: PMCPMC2845052.

7. Bello-Chavolla OY, Antonio-Villa NE, Vargas-Vazquez A, Viveros-Ruiz TL, Almeda-Valdes P, Gomez-Velasco D, et al. Metabolic Score for Visceral Fat (METS-VF), a novel estimator of intra-abdominal fat content and cardio-metabolic health. Clin Nutr. 2020;39(5):1613-21. Epub 20190730. doi: 10.1016/j.clnu.2019.07.012. PubMed PMID: 31400997.
